# Effect of Body Mass Index on respiratory parameters: A cross-sectional analytical Study

**DOI:** 10.12669/pjms.35.6.746

**Published:** 2019

**Authors:** Urooj Bhatti, Zulfiqar Ali Laghari, Binafsha Manzoor Syed

**Affiliations:** 1 Dr. Urooj Bhatti, Lecturer, Department of Physiology, Liaquat University of Medical and Health Sciences (LUMHS), Jamshoro, Sindh, Pakistan; 2 Prof. Dr. Zulfiqar Ali Laghari, PhD. Professor of Physiology, University of Sindh, Jamshoro, Pakistan. Lecturer, Department of Physiology, Liaquat University of Medical and Health Sciences (LUMHS), Jamshoro, Sindh, Pakistan; 3 Dr. Binafsha Manzoor Syed, MBBS, PhD. Director ORIC Medical Research Centre, Liaquat University of Medical and Health Sciences (LUMHS), Jamshoro, Sindh, Pakistan

**Keywords:** Body Mass Index, Healthy adults, Respiratory parameters

## Abstract

**Objective:**

To assess association of Body mass index (BMI) on respiratory parameters by performing spirometry in apparently healthy adults living in the district Jamshoro and Hyderabad, Sindh, Pakistan.

**Methods:**

A cross sectional study was conducted at Department of Physiology, Liaquat University of Medical and Health Sciences Jamshoro, Pakistan from January to September 2015. A total of 180 underweight, normal, overweight and obese participants, aged between 18 to 40 years were included in the study. BMI was calculated by measuring weight and height by BMI scale (RGZ-160) in standing position. Pulmonary parameters were determined by spirometry on Power lab (AD instruments). Pulmonary parameters were compared between subjects in different categories of BMI.

**Results:**

Mean age of participants was 21.83±5.88 years and the mean BMI was 25.10±6.55 kg/m^2^. The study results revealed that except for FVC, which was not statistically significant (p=0.45) all other respiratory parameters were significantly different (p≤0.05) in all BMI categories. Mean FEV1/FVC ratio (93.1 vs. 90.3, 86.4 and 86.6 respectively) was highest among underweight as compared to overweight, obese and normal weight individuals. The mean VT was 1.22 vs. 0.90, 1.01 and 0.84 respectively, IRV was 1.04 vs. 1.18, 1.23 and 1.20 respectively, IC was 2.26 vs. 2.08, 2.25 and 2.05 respectively, VC was 2.63 vs. 2.42, 2.54 and 2.54 respectively, TLC was 2.98 vs. 3.03 vs. 3.18 and 3.17 respectively among underweight, overweight, obese and normal weight participants.

**Conclusion:**

We found a significant association between body mass index and pulmonary function parameters. Obesity causes detrimental effects on respiratory system.

## INTRODUCTION

According to World Health Organization (WHO) classification for Asian people; Body Mass Index (BMI) can be used to classify obesity and overweight. For the calculation of BMI, weight (Kg) is divided by height (meter square). If BMI is 23-24.9 Kg/m^2^, it is overweight at risk and if BMI ≥25 Kg/m^2^ then it is labelled as obesity. Research studies have shown that about 1.5 million persons above the age of 20 years are overweight while 10% are obese.^1^ Lung function decreases as BMI increases. Normal BMI is associated with normal forced vital capacity and forced expiratory volume in one second (FEV_1_).^2^ Lung function parameters including lung volumes and respiratory efficiency have been reported to be abnormal in obese persons.^3^

Weight affects respiratory parameters because it causes small airway dysfunction, expiratory flow limitation, respiratory mechanics change, chest wall and lung compliance reductions, decreased respiratory muscle strength, decreased pulmonary gas exchange, lower breathing control and limitations in exercise capacity.^4^

Impairment of respiratory function occurs and lung expansion decreases if the fat deposits over diaphragm, abdomen and intercostal muscles. It leads to decreased functional residual capacity (FRC), expiratory reserve volume (ERV), forced expiratory reserve volume in 1sec (FEV1), and total lung capacity (TLC).^5^ Generally vital capacity (VC) and total lung capacity (TLC) remains normal but it may be decreased by ≥30%, if obesity is severe. There is an increased breathing effort, if there is “abnormal chest wall resistance” or if there is increased airway resistance.^6^ It is seen that if BMI is between 20 and 30kg/m^2^ then the changes in FRC and ERV are quite similar and there is no big difference in these BMI groups.^7^

Most of the Previous local and international studies were lacking the healthy population in their studies as they were done on obese persons and observed respiratory variables in sick obese persons only whereas in our study we also included healthy people i.e. persons having different BMI including those with normal BMI and observed respiratory variables in apparently healthy obese persons. However, there is limited data available on Pakistani population.

## METHODS

A cross sectional study was done at Department of Physiology, Liaquat University of Medical and Health Sciences Jamshoro, Pakistan from January to September 2015. The study protocol was approved by the hospital Ethics Committee.(N. JUIMHS/REC/114 December 4, 2013) A total of 180 underweight, normal, overweight and obese participants aged between18 to 40 years were included in the study using non-probability purposive sampling technique. The sampling was purposive for selection of specified population based on their body weight. The selection of obese and underweight was deviant purposive technique.

Sample size was calculated by using open epi sample size calculator version 3.01. The parameters included were mean and standard deviation of FEV, 108±1.2 of the subjects having duration of obesity less than five years and 107±1.2 of subjects having duration of obesity more five-ten years,^8^ The 95% confidence interval and 90% power was considered for sample size calculation. Minimum needed sample size came up as 31 participants in each group of BMI category. A total of 180 participants were calculated.

### Protocol

All the participants included were non-smokers, without any known pulmonary, cardiac or chest deformities and not worked in dust containing environment. For the recruitment of participant’s, volunteer’s recruitment posters were posted at different locations at LUMHS hospital and university campus. After fulfilling inclusion criteria i.e., age between 18-40 years, males and non-pregnant females, non-smokers and those who don’t have any respiratory disorder like pulmonary Koch’s or reactive airway disease and the exclusion criteria were pregnant females, smokers, persons having respiratory and/or cardiac disease, written informed consent was taken from the participants. Before medical screening session five minutes’ rest was given to every participant. After recruitment of volunteers, they were advised not to take heavy meals tea or coffee two hours before procedure. For the exclusion of any cardiac disorder, Blood pressure was taken in sitting position, from right arm with the help of the aneroid sphygmomanometer (Yamasu Japan) and stethoscope (Littman Company) and also electrocardiogram was taken by ECG (FK 12) machine.

Extra clothing and shoes of the participants were removed before taking BMI on scale (RGZ-160, China). Height was recorded by (RGZ-160, China) in cm (with range of 70-190 cm), with bare feet. Weight was recorded in kg using a mechanical scale (RGZ-160, China) with a capacity of 160 kg Following formula was used for BMI calculation:

i.e., weight in kg/height in meter^2^ (cm is converted into m^2^).

Spirometry was done on Power lab (model 15T AD instrument) used for measuring respiratory parameters. Normal tidal breathing was noted for one-minute duration. For recording tidal breathing, participants were instructed to breathe in maximally and then breathe out. Lab chart on Power lab software was used for the collection and saving of data that was later on exported to the MS Excel for analysis. Lab chart software was used in case, if respiratory parameters not determined by the Power lab.

### Data analysis

SPSS (version 23.0, Armonk, NY: IBM Corp.) was used for data analysis. Frequencies and percentage were calculated for categorical variables such as age groups, gender, body mass index groups and history of pulmonary problems, hypertension, and diabetes in family. Means and standard deviations were calculated for continuous variables like age in years, weight, height, body mass index and spirometric parameters. Kruskal Wallis H test was applied to compare the means of pulmonary parameters across the categories of BMI. P-value ≤0.05 was considered as statistically significant. Generalized linear model (GLM) was used to estimate the parameters of linear regression model and measure the effect of body mass index on respiratory parameters.

## RESULTS

A total of one hundred and eighty (180) participants were included in the study for analysis. No participant left the study so total 180 participants completed the study. Out of which forty (40) were normal weight (18.5-22.9kg/ m^2^), thirty four (34) were underweight (<18.5 kg/ m^2^), seventeen (17) were overweight at risk (23-24.9 kg/ m^2^), fifty five (55) were obese 1(25-29.9 kg/ m^2^) and forty four (44) were obese 2(≥30 kg/ m^2^). The study results revealed that the mean age of the study participants was 21.83 (SD=±5.88) years whereas 52.2% of them were males. Their mean BMI was25.10(SD±6.55 kg/m^2^) whereas a majority of them were either overweight (i.e. 32.2%) or obese (i.e. 26.1%) (Table-I).

**Table I:** Baseline Information of Studied Samples (n=180).

Characteristics		N	%
Age(years)	Mean, SD	21.83	5.88
Gender	Male	94	52.2
	Female	86	47.8
BMI (kg/m2)	Mean, SD	25.10	6.55
BMI Group*	Underweight (<18.5)	34	18.9
	Normal weight (18.5-22.9)	30	16.7
	Overweight at risk (23-24.9)	17	9.4
	Obese 1 (25-29.9)	55	30.6
	Obese 2 (≥30)	44	24.4
Family History of Diabetes	Yes	81	40
Family History of Hypertension	Yes	72	45
Family History of Respiratory Disease	Yes	30	15

* World Health Organization, Regional office for the western pacific, International association for the study of obesity. International obesity task force. The Asia pacific prospective: redefining obesity and its treatment. Melbourne, Health communication Australia, 2000.

While assessing the association of BMI with pulmonary function parameters by performing spirometry it was observed that BMI has insignificant impact on FVC(L) and ERV(L) (p= 0.69 & 0.15 respectively) where as it has significant (p<0.01) impact on FEV1 (L), FEV1 / FVC (%), Average VT (L), IRV(L), IC(L), VC(L), TLC(L), FRC(L). (Table-II)

**Table II:** Impact of Body Mass Index on Pulmonary Function Test by Performing Spirometry.

Pulmonary Function Parameters	Body Mass Index										p-value

	Underweight (n=34)		Normal weight (n=30)		Overweight at risk (n=17)		Obese 1 (n=55)		Obese 2 (n=44)		

	Mean	SD	Mean	SD	Mean	SD	Mean	SD	Mean	SD	
FVC (L)	2.27	0.48	2.37	0.65	2.47	0.68	2.28	0.58	2.49	0.75	0.69
FEV1 (L)	2.38	0.74	3.48	0.68	3.31	0.97	1.98	0.52	2.07	0.55	<0.01*
FEV1 / FVC (%)	93.1	4.04	86.6	7.70	90.3	7.48	86.4	10.4	87.1	8.25	<0.01*
Average VT (L)	1.22	0.42	0.84	0.22	0.90	0.29	1.01	0.60	1.62	0.50	<0.01*
IRV	1.04	0.21	1.20	0.49	1.18	0.42	1.23	0.41	1.64	0.57	<0.01*
ERV	0.36	0.22	0.48	0.40	0.33	0.16	0.29	0.16	0.37	0.24	0.15
IC	2.26	0.45	2.05	0.55	2.08	0.60	2.25	0.87	3.26	0.75	<0.01*
VC	2.63	0.44	2.54	0.83	2.42	0.65	2.54	0.88	3.64	0.75	<0.01*
TLC	2.98	0.49	3.17	1.03	3.03	0.82	3.18	1.10	4.55	0.94	<0.01*
FRC	0.65	0.25	1.12	0.58	0.94	0.27	0.92	0.29	1.28	0.33	<0.01*

* p<0.05 was considered significant using Kruskal Wallis test.

The results of generalized linear model (GLM) to measure the effects of body mass index on respiratory parameters showed that BMI is negatively associated with FEV1 (L), FEV1/FVC (%), and positively associated with remaining respiratory parameters (p<0.05). However there was no significant association of BMI with FVC(L) and ERV(L) (Table-III).

**Table III:** Effect of Body Mass Index (BMI) on respiratory parameters using generalized linear model (GLM).

Dependent Variables	Independent Variable	Beta	Standard Error	p-value	95% Confidence Interval	

					Lower Bound	Upper Bound
FVC (L)	BMI	0.009	0.007	0.206	-0.005	0.024
FEV1 (L)	BMI	-0.036	0.01	<0.01*	-0.055	-0.017
FEV1 / FVC (%)	BMI	-0.354	0.096	<0.01*	-0.543	-0.165
Average VT (L)	BMI	0.022	0.006	<0.01*	0.01	0.035
IRV	BMI	0.031	0.005	<0.01*	0.021	0.041
ERV	BMI	-0.003	0.003	0.257	-0.009	0.002
IC	BMI	0.053	0.009	<0.01*	0.036	0.071
VC	BMI	0.05	0.01	<0.01*	0.031	0.069
TLC	BMI	0.079	0.012	<0.01*	0.056	0.102
FRC	BMI	0.026	0.005	<0.01*	0.017	0.035

* p<0.05 was considered significant.

## DISCUSSION

The study was done to assess the impact of BMI on pulmonary function test in young apparently healthy adults. Our study results are consistent with findings of Banerjee et al., 2014 which showed no significant difference in the mean of FVC across BMI groups. In contrast to FVC, the mean value of FEV1 was significantly different across groups of body mass index. There was negative association between obese, overweight and underweight subjects and FEV1, means as BMI increases there is decrease in FEV1. Our results revealed that FEV1/FVC ratio was also significantly different across BMI groups. There was positive association with decreasing BMI. Moreover, in similar study FEV1/FVC ratio had significant positive relation with BMI ,that is FEV1/FVC ratio was affected by increasing BMI.^9^ In another study, FEV1and FVC was lower in females as compared to males, even though the participants belonged to different age groups but the results are comparable to our study.^10^ Another earlier study showed that BMI had no significant relationship with any of the pulmonary function parameters among obese/overweight children; however, ‘normal weight’ group had lower FEV1 and FVC values.^11^ This difference may be due to different ages of the study population as that study only included school going children.

In our study there was negative association between FVC and overweight subjects, while there was positive association between FVC and Obesity. FEV1 had negative relationship with overweight and obesity. Regarding FEV/FVC ratio there was positive correspondence with underweight subjects; there was negative connection with overweight, while there was no association with obesity. In an earlier Pakistani local study BMI values had significant effect on lung volumes; there was a trend of decreasing mean FVC and FEV1 with increasing BMI though FEV1/FVC had no significant association with BMI. Lung volumes including FVC and FEV1 had significant negative association with BMI in both genders though males had stronger association than females.^12^ In another study, a significant reduction in the pulmonary functions was observed in case of the obese subjects in comparison to the subjects exhibiting normal BMI, BMI was found to have negative association with pulmonary parameters. FVC was decreased in overweight subjects, while it was almost same in overweight and underweight subjects.^13^ In yet another study, the normal pattern of pulmonary function decreased with increasing BMI and obese group had significantly lower pulmonary function as compared to normal group.^14^ Another similar study showed FVC and FEV1 to significantly decrease in the overweight group as compared to the normal control group.^15^ Results of our study showed that the mean values of FEV1/FVC ratio were significantly different between underweight and overweight, obese and normal weight groups as it was significantly higher in underweight group as compared to other body mass index groups. In another study FEV1/FVC ratio was found to have no significant association with overweight, while there was a negative relation with obesity.^14^

Another study showed a positive relationship between FVC, FEV1 and underweight, while FEV1 and BMI were negatively interrelated with normal weight people. In overweight males FVC, FEV1 were negatively associated with BMI. FVC and FEV1 was positively connected with BMI/body fat percentage in underweight females, while there was a significant positive association between BMI and FVC in normal weight females, correlation was also positive in overweight females.^16^

In our study there was a negative relationship between ERV and underweight, overweight and obese categories of BMI. ERV was found to be significantly low in overweight as compared to normal and obese subjects. In another study ERV was also reported to have a negative association with overweight and obesity. According to Koenig, 2001 this decrease in ERV is due to reduced diaphragmatic mobility, as increased abdominal volume of obese person’s cause’s upward pressure of diaphragm which that causes decreased thoracic cavity diameter; also decline in ERV causes obstruction of small airways and rise residual volume leading to reduction in gas exchange.^17^

Our study revealed that tidal volume (VT) was found to be significantly higher among obese as compared to other BMI groups. A study of Costa (2008) showed dissimilar findings that VT was similar in obese and normal persons; may be due to different spirometers were used. Our results showed that there was a positive association between (higher) IRV and overweight and obese categories of BMI, while there was a negative link between (lower) IRV and underweight category of BMI. In a similar study IRV had a moderate positive association with obesity.^18^ Obesity causes deleterious effect on ventilatory mechanics, probably due to lung compression which causes a reduction in ERV, leading to a compensatory increase in IRV in attempt to maintain a constant VC. In line with our results an earlier study also reported a positive association between obesity and IRV.^19^

As Inspiratory capacity(IC) is equal to VT plus IRV (L) it remained higher in obese in our study (Table-II). Because VT and IRV both are increased in obese in our results. The mean values of IC were significantly different between underweight and obese subjects (Table-III). An earlier study also reported that IC was positively associated with Obesity.^20^ As vital capacities is equal to IRV + ERV + VT (L); The mean values of VC were significantly higher in obese than in all other groups. In another study VC was reported to have a negative relationship with BMI.^21^ An earlier study did not report any relation of VC with normal weight and obesity.^18^

Unlike other studies in our study TLC (maximum air which can be inspired) was found to be significantly higher in obese while there was no significant relation between TLC and overweight and underweight subjects. These results are inconsistent to this study in which TLC was reported to have negative association with overweight and obese persons because of decrease in compliance due weight. Moreover, FRC was found to be significantly higher in obese as compared to other groups, however mean FRC was found to be similar between overweight and normal weight subjects. In another study FRC was reported to have a significant negative association with overweight and obesity.^17^ Difference in results may be due to difference in methodology and sample size and this is the first study in which TLC and FRC both capacities are calculated as they included residual volume (RV) amount of air remains in lung after maximum expiration, because on spirometry we cannot measure RV here we have determined

RV by using equation 1, which determines the predicted RV value for the volunteer. This equation predicts RV for 16–34 year-old subjects of either sex (Gaensler and Wright, 1966).

Equation 1: RV = predicted VC X 0.25 (L)

Consistent with our study results Kohli PG, 2017 also showed that FVC, FEV1, IRV, ERV and FRC had significantly different mean values among male and female subjects where they were significantly higher in males as compared to females.^13^ May be males have more respiratory muscle strength and higher compliance due more surface area for gas exchange because males are taller than females and height is robust cause which effects pulmonary function tests.^13^,^22^

## CONCLUSION

We found a significant association between body mass index and pulmonary function parameters. Obesity has detrimental influence on respiratory physiology of healthy persons as well.

### Authors’ Contribution:

This study was conducted as part of PhD project of UB, ZL and BMS are PhD Supervisors of **UB**

**UB:** Conceived, designed and did statistical analysis, manuscript writing, data collection, is responsible for integrity of research.

**ZL:** Supervised project, did review and final approval, editing of manuscript.

**BMS**: Supervised project, did review and final approval, editing of manuscript.
